# Obsessive-compulsive symptoms and resting-state functional characteristics in pre-adolescent children from the general population

**DOI:** 10.1007/s11682-022-00732-8

**Published:** 2022-11-02

**Authors:** Cees J. Weeland, Odile A. van den Heuvel, T. White, H. Tiemeier, C. Vriend

**Affiliations:** 1 Department of Anatomy and Neurosciences, Department of Psychiatry, Amsterdam UMC, Vrije Universiteit Amsterdam, PO Box 7057, 1007 MB De Boelelaan 1117, 1081HV, Amsterdam, Netherlands; 2 Dept. Child and Adolescent Psychiatry/Psychology, Erasmus Medical Center, Wytemaweg 8, 3015 GD Rotterdam, the Netherlands; 3 The Generation R Study Group, Erasmus Medical Center, Doctor Molewaterplein 40, 3015 GD Rotterdam, the Netherlands; 4 Harvard TH Chan School of Public Health, 677 Huntington Ave, 02115 Boston, MA USA

**Keywords:** Obsessive-compulsive disorder, Neuroimaging, Functional MRI (fMRI), Developmental epidemiology, Neural networks.

## Abstract

While functional brain characteristics of obsessive-compulsive disorder have been extensively studied, literature on network topology and subnetwork connectivity related to obsessive-compulsive symptoms (OCS) is sparse. Here we investigated the functional brain characteristics of OCS in children from the general population using a multiscale approach. Since we previously observed OCS-related differences in thalamus morphology, we also focused on the network participation of thalamic subregions. The study included 1701 participants (9–12 years) from the population-based Generation R study. OCS were measured using the Short Obsessive-Compulsive Disorder Screener. We studied the brain network at multiple scales: global network topology, subnetwork connectivity and network participation of thalamic nodes (pre-registration: https://osf.io/azr9c). Modularity, small-worldness and average participation coefficient were calculated on the global scale. We used a data-driven consensus community approach to extract a partition of five subnetworks involving thalamic subregions and calculate the within- and between-subnetwork functional connectivity and topology. Multiple linear regression models were fitted to model the relationship between OCS and functional brain measures. No significant associations were found when using our preregistered definition of probable OCS. However, post-hoc analyses showed that children endorsing at least one OCS (compared with controls) had higher modularity, lower connectivity between frontoparietal, limbic and visual networks as well as altered participation of the lateral prefrontal thalamus node. Our results suggest that network characteristics of OCS in children from the general population are partly symptom-specific and severity-dependent. Thorough assessment of symptom dimensions can deepen our understanding of OCS-related brain networks.

**Supplementary Information:**

The online version contains supplementary material available at 10.1007/s11682-022-00732-8.

## Introduction

Functional and structural imaging studies of obsessive-compulsive disorder (OCD) have implicated dysfunction of cortico-striato-thalamo-cortical (CSTC), fronto-limbic, fronto-parietal and cerebellar circuity in its pathophysiology (Shephard et al., 2021). Obsessive-compulsive symptoms (OCS) are also common in the general population and are predictive of developing OCD in the future (Fullana et al., 2009). Studying the neural correlates of OCS and OCD at this early stage may provide clues to the pathophysiology from subclinical to clinical OCD. Also, insight into the underlying neurocircuitry of OCD may lead towards more precise treatment strategies targeting specific circuits. Mega-analyses on morphometry have shown that OCD patients exhibit robust age-dependent subcortical differences, with larger thalamus volume observed in pediatric patients and smaller volume in adults patients (Weeland Kasprzak, et al., 2022). We recently showed that children from a community-based sample with clinical-level OCS (i.e. ‘probable OCD’ cases) also display larger thalamus volume compared with symptom-free peers (Weeland et al., 2021), with possible differentiation across thalamic subregions (Weeland, Vriend, et al., 2022). However, it is not clear whether these volumetric differences also translate to altered functional connectivity.

Given that the brain is organized as a network in which the thalamus forms an important hub connecting subcortical and cortical regions (McFarland & Haber, 2002; van den Heuvel & Sporns, 2011), OCS-related functional differences of the thalamus may impact network characteristics of the brain on the global, subnetwork and local level. This notion is largely supported by the literature on functional characteristics of OCD, even though this predominantly includes clinical studies in adults investigating pairwise connectivity patterns. In a recent meta-analysis, OCD-related functional connectivity differences have been found between and within various intrinsic brain networks, including the frontoparietal, salience and limbic network, as well as between the frontoparietal network and mediodorsal thalamus (Gürsel et al., 2018). Pertaining to the thalamus, OCD patients show altered connectivity between the thalamus and subcortical and widespread cortical regions (Fitzgerald et al., 2011; Li et al., 2019; Sha et al., 2020; Stern et al., 2012), further supporting that clinical OCD is associated with widespread connectivity alterations that also involve the thalamus. Studies investigating OCS-related functional characteristics in the general population are sparser. Suñol and colleagues found that subclinical OCS severity in children aged 8–12 years is associated with global dysconnectivity of the left ventral putamen and medial dorsal thalamus. Also, specific symptom dimensions showed differential patterns of functional connectivity across limbic, sensorimotor and insular regions (Suñol et al., 2021). In 8–21 year old youths from the general population, Alexander-Bloch et al. found global connectivity differences related to OCS, involving dorsal and ventral attention, default mode and frontoparietal network (Alexander-Bloch et al., 2021). This partly aligns with another study that found that OCS in 9–10 year old children was associated with alterations in the connectivity of subnetworks that include the dorsal attention, ventral attention and default mode network (Pagliaccio et al., 2021).

Taken together, the literature provides tentative evidence for OCD- and OCS-related widespread brain connectivity alterations, which include well-known functional subnetworks as well as specific thalamic regions but the evidence is sparse. Although clinical studies have found OCD-related differences in global network organization such as decreased small-worldness and modularity (Armstrong et al., 2016; Zhang et al., 2011), no subclinical OCS studies in children have looked at global network organization. Furthermore, no previous studies have investigated theparticipation of the thalamus within the brain network.

Network organization of the brain can be quantified using a graph theoretical approach, which defines the brain as a network where brain regions form the ‘nodes’ of the network and connections between regions are defined as ‘edges’. From these nodes and edges, various measures can be computed that describe organizational features of the network. Graph theory can be used to describe the organization of a network as a whole or the characteristics or participation of individual nodes within the network. Given the importance of the thalamus for the information flow across the brain network, we hypothesized that the previously reported OCS-related alterations in thalamus morphology may also affect its function and lead to perturbations in network organization across the global, subnetwork and local (thalamus) scale. We used a graph theoretical approach to quantify network organization at the global and local level (see Methods section on Graph Measures for details).

We therefore applied a multiscale approach to investigate the functional network characteristics of OCS in children from the population-based Generation R Study. Since subnetwork parcellations provided by the literature are mostly derived from adults and do not include thalamic subnuclei, we applied a data-driven approach on our pediatric sample to parcellate the Brainnetome Atlas into separate modules for our subnetwork analysis (Bassett et al., 2013; Fan et al., 2016). We hypothesized that OCS in children from the general population is associated with differences in (1) global network topology, (2) functional connectivity between brain modules that include thalamic subnuclei, and (3) the participation of specific thalamic nodes in network communication.

## Methods

### Study design

The current study is embedded within the Generation R Study, a prospective population-based birth cohort in Rotterdam, the Netherlands (Kooijman et al., 2016). As part of the cohort’s MRI study, 3992 children aged 9–12 years underwent a brain MRI scan between March 2013 and November 2015 (White et al., 2018). Participants with dental braces, incomplete resting-state functional MRI (RS-fMRI) scans, or major incidental findings were excluded (Jansen et al., 2017). To limit the impact of head motion, we excluded children with a mean framewise displacement above 0.25 mm or with more than 20% of volumes with a framewise displacement above 0.2 mm. Further exclusions were incomplete data on OCS (see below), registration errors or insufficient signal in nodes of the neuroanatomical atlas.

### Obsessive-compulsive symptoms

OCS were measured using the Short Obsessive-Compulsive Disorder Screener (SOCS) rated by the primary caregiver (Uher et al., 2007). SOCS measures compulsive symptoms with seven-items on a 3-point Likert scale, with a cut-off score of six or higher that has excellent test characteristics (Uher et al., 2007). Consistent with our previous studies (Weeland, Vriend, et al., 2022; Weeland et al., 2021), we used this cut-off to define a ‘probable OCD’ group for case-control analyses and used continuous sum scores to investigate linear associations. We allowed a maximum of one missing item (< 25%), in which case we calculated weighted sumscores [sum of 6 items * (7 / 6)].

### Image processing

MRI data was acquired on a 3T GE Discovery MR750w scanner (Milwaukee, WI, USA) using a standard 8-channel head coil. We obtained structural T1-weighted images using an inversion recovery fast spoiled gradient-recalled echo sequence (IR-FSPGR). Echo planar imaging was used to acquire 200 volumes of RS-fMRI data [TR = 1,760 ms, TE = 30 ms, flip angle = 85°, matrix = 64 × 64, FOV = 230 mm × 230 mm, slice thickness = 4 mm]. One scanning run per participants was acquired that lasted 5 min and 52 s per scan. The resting state images were preprocessed using the standardized *fMRIPrep* (v20.1.1) pipeline, that includes head motion correction, slice-timing correction and ICA-AROMA to remove motion artifacts (Esteban et al., 2019; Pruim et al., 2015). The first four non-steady state volumes were removed from the functional images. The remaining volumes were spatially smoothed by applying a 6 mm full-width half-maximum (FWHM) isotropic Gaussian kernel. We applied simultaneous nuisance regression and temporal filtering at 0.009 to 0.08 Hz using the Denoiser tool (https://github.com/arielletambini/denoiser). Nuisance regression was performed by using the ICA-AROMA components and eight physiological regressors: mean signal in the white matter and cerebrospinal fluid, their squares, temporal derivatives and derivatives squared. Aggressive denoising was applied in ICA-AROMA, which involves regressing out all variance associated with noise components, including shared variance with signal components. The functional scans were registered to the anatomical T_1_-weighted images and we parcellated the anatomical scan into 246 nodes using the Brainnetome atlas, which contains the caudal temporal, rostral temporal, medial pre-frontal, lateral pre-frontal, pre-motor, posterior parietal, occipital and sensory thalamus parcellations (Fan et al., 2016). We discarded nodes if they comprised less than four signal-containing voxels, resulting in the exclusion of four nodes of the Brainnetome atlas across the entire sample (left and right area TI, corresponding to the temporal agranular insular cortex; left and right area A28/24, corresponding to the entorhinal cortex). Therefore, the resulting network of each participant consisted of 242 nodes. We constructed connectivity matrices by calculating absolutized Pearson correlation coefficients of the time series between each pair of nodes. To evaluate how the anatomical regions defined by the Brainnetome atlas are functionally connected across the network, we applied a data-driven consensus community algorithm to partition the brain into five communities (or subnetworks) with large resemblance to existing resting-state networks such as those found by Yeo and colleagues (for details see Appendix S1). ***nodes and edges, various measures***.

### Graph measures

**Fig. 1:**
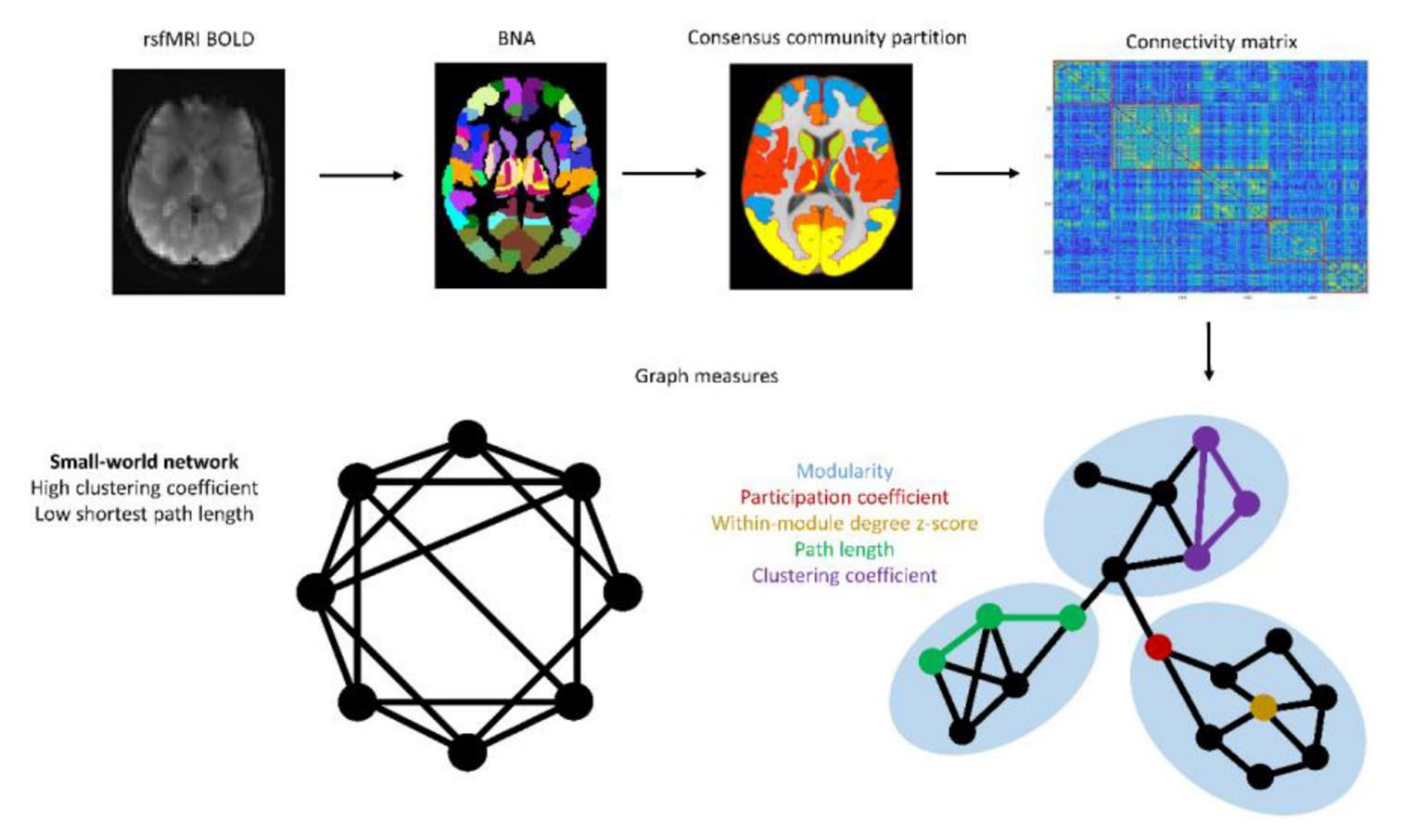
Visual representation of image processing steps and graph measures. Visual representation of steps in the calculation of the graph measures. These steps include the extraction of timeseries, warping to the Brainnetome atlas (BNA), construction of the connectivity matrix and consensus community partition and calculation of graph measures. Abbreviations: BNA = Brainnetome Atlas; BOLD = Blood-oxygen-level-dependent; rsfMRI = resting-state functional magnetic resonance imaging

The global graph measures calculated for analyses were average participation coefficient (PC), modularity and small-worldness based on weighted connectivity matrices (see Fig. 1 for a visual representation). PC is a measure of the distribution of an individual node’s connections across the different communities of a graph. The global (i.e. whole-brain) average of this metric for all nodes in a graph represents the average PC. Modularity quantifies the degree to which a network can be divided into segregated modules (or communities): groups of nodes with stronger connections among each other than with other nodes in the network. The brain is considered to have a small-world network organization, which is characterized by high local clustering and high efficiency. Clustering is expressed by the clustering coefficient, which expresses the fraction of a node’s neighbors that are also neighbor’s of each other. Efficiency captures the ease by which information can travel through a network. Global efficiency is derived from the average path length between two nodes in a network. Small-worldness of the global network is quantified by comparing the local clustering and average path length of a graph to that of a similar random network. The within-module degree Z score of a node represents the strength of its connections with other nodes within its module. To quantify the participation of individual thalamic nodes in the network, we calculated the PC and within-module degree Z-score for the eight thalamic nodes within the graph. The left and right side of seven out of the eight thalamic nodes were assigned to the same community in the consensus community partition. We calculated the bilateral mean of the thalamic graph measure for these nodes. For the caudal temporal node, the only node where left and right were assigned to different communities, we computed the graph metrics separately. Therefore, we compared nine different node values and corrected for multiple comparisons accordingly.

**Fig. 2:**
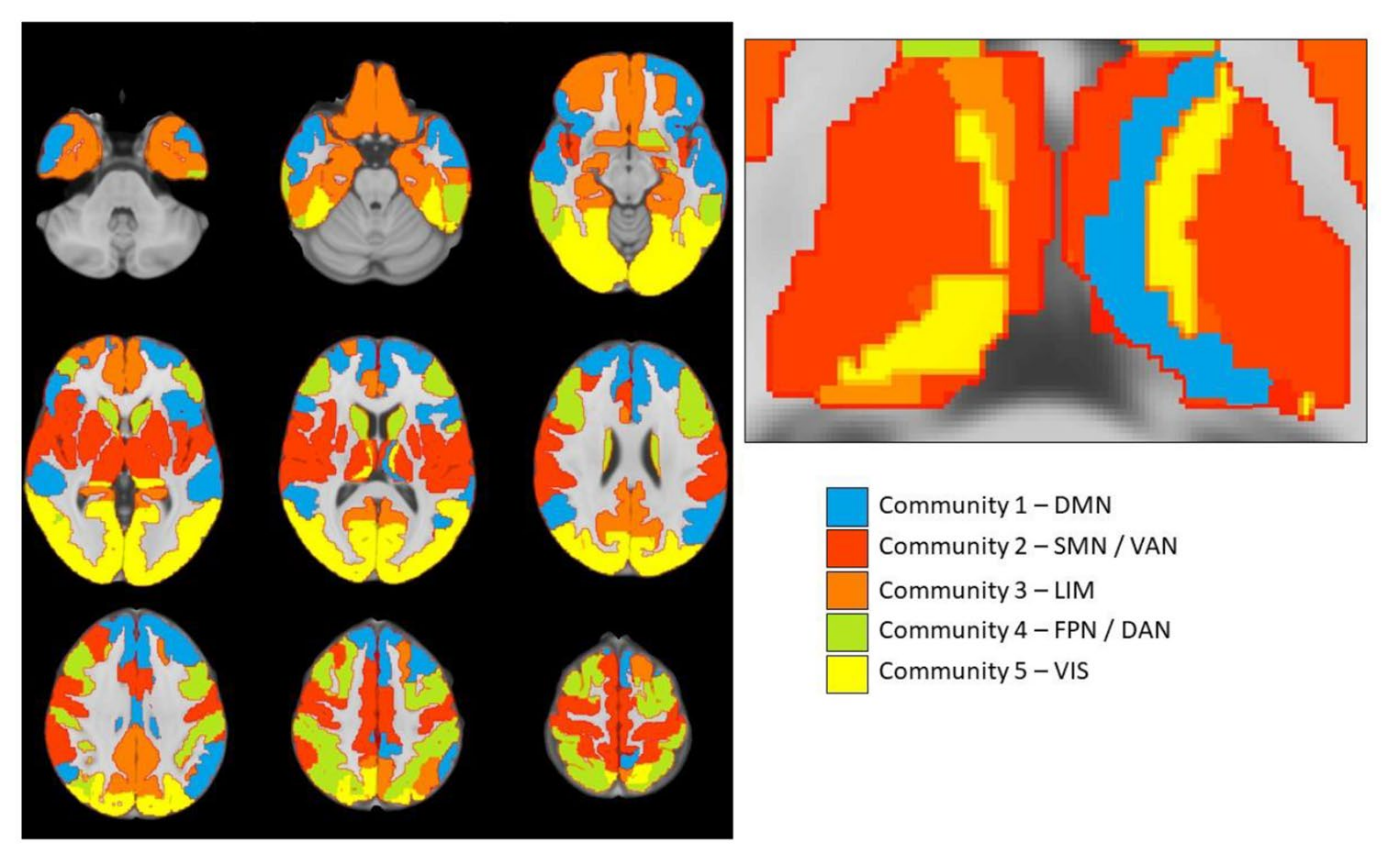
Axial view of data-driven consensus community partition of resting-state functional connectivity subnetworks. (Note: Consensus partition at a resolution parameter *gamma* of 1.05. The right figure shows a close-up of the partition across thalamic subregions. Abbreviations: DMN = Default Mode Network; SMN / VAN = Somatomotor Network / Ventral Attention Network; LIM = Limbic Network; FPN / DAN = Frontoparietal Network / Dorsal Attention Network; VIS = Visual Network)

### Statistical analysis

We preregistered our analysis plan prior to conducting any analyses (https://osf.io/azr9c). All statistical analyses were performed in R, version 4.0.3 (R Core Team, 2020). We report descriptive statistics of the variables and covariates of interest. We performed a continuous analysis and case-control comparison between ‘probable OCD’ and symptom-free controls of the global graph measures, between-subnetwork connectivity, and node-specific graph measures. Multiple linear regression models were used with OCS sumscores as continuous predictor and probable OCD as a binary independent variable in the case-control analysis. All models were adjusted for age, sex and maternal ethnicity. To control for concurrent behavioral and emotional problems, models were additionally adjusted for sum score on the Child Behavior Checklist (CBCL) minus the items of the CBCL-OCS subscale to avoid overcorrection (Achenbach & Rescorla, 2001; Nelson et al., 2001). Regression models were bootstrapped using 10,000 iterations to obtain reliable estimates. We performed multiple comparisons correction on the subnetwork level for each parallel analysis (10 between-community tests) and on the node level for the nine thalamic nodes that were tested by applying a Benjamini-Hochberg False Discovery Rate correction (q < 0.05). Missing values of ethnicity were imputed with multiple imputation using chained equations (*mice* package, R) (van Buuren & Groothuis-Oudshoorn, 2011). We performed a complementary analysis using network-based statistics to identify clusters of brain regions that are significantly different between probable OCD controls (details are described in Appendix S3). As a posthoc analysis, we defined a binary ‘high OCS’ variable to compare children scoring ‘often’ on at least one OCS item (n = 133) with the remaining group of children (n = 1568) that scored only ‘sometimes’ or ‘never’ on all items (details in Appendix S4). We also conducted a posthoc continuous analyses using the CBCL-OCS subscale as variable of interest.

## Results

### Sample characteristics

Of the initial sample of 3992, we excluded participants based on predetermined criteria (see flowchart in Figure S1), leading to a final sample of 1701 participants. Sample characteristics are displayed in Table 1.

**Table 1:** Sample Characteristics

	Whole Sample (N = 1701)	Probable OCD Cases (N = 109)	Controls (N = 767)
**Child characteristics**			
Age at MRI	10.1 (± 0.6)	10.2 (± 0.6)	10.1 (± 0.6)
Sex (% boys)	49.2%	46.8%	48.0%
Non-verbal IQ	104.6 (± 14.9)	99.0 (± 15.1)	106.1 (± 14.1)
CBCL^a^	14.9 (± 14.0)	24.2 (± 20.2)	11.5 (± 11.1)
SOCS	1.67 (± 2.11)	7.1 (± 1.3)	0 (0)
**Maternal Characteristics**			
Ethnicity			
*Dutch*	1114	36	576
*Non-Dutch Western*	223	11	100
*Non-Dutch Non-Western*	350	61	86
*Missing*	14	1	5
Maternal age at birth	31.8 (± 4.6)	30.5 (± 5.3)	30.6 (± 5.0)

Note: CBCL = Child Behavior Checklist; MRI = Magnetic Resonance Imaging; SOCS = Short Obsessive-Compulsive Disorder Screener. ^a^CBCL score minus CBCL-OCS subscale items

### Pre-registered analyses

We found no significant associations between OCS and average PC, modularity and small-worldness in either continuous or case-control analyses (Table S1). Distributions of the graph measures and subnetwork functional connectivity are presented Tables S2-S4 and Figures S2-S4). In the subnetwork analyses, no significant associations were found between OCS and subnetwork connectivity in the continuous (Table S5) and case-control analyses (Table S6). The thalamic node analyses did not show any significant associations between OCS and thalamic node PC or within-module degree Z-score in the continuous (Table S7-S8) or case-control analyses (Table S9-S10). Results were similar when repeating the analyses with individuals with a SOCS > 0 (data not shown). Finally, in our NBS analysis no significant components between the probable OCD and control groups.

### Post-hoc analysis of ‘high OCS’ children scoring high on at least one symptom

Children with high OCS (n = 133) compared with controls (n = 1568) had higher modularity compared with controls (B = 0.0005, p = 0.008) (Table 2, significant results), lower connectivity between the frontoparietal-dorsal attention network (FPN/DAN) and visual network (VIS) (B = -0.015, p = 0.02) ) and lower connectivity between the FPN-DAN and limbic network (LIM) (B= -0.012, p = 0.02). Finally, high OCS was associated with lower within-module degree z-score of the lateral prefrontal thalamus node (B = -0.118, P = 0.03). We compared the ‘probable OCD’ group, ‘high OCS’ group and remaining controls on demographic characteristics, OCS scores and CBCL subscale scores (Table S11). The high OCS group compared with the probable OCD group had a significantly larger proportion of children scoring high on the hand cleaning item of the SOCS (χ^2^ = 7.60, *p* = 0.0058). Excessive hand washing therefore seems to drive the results. Indeed, children that scored high on the hand washing item had significantly higher modularity (B = 0.01, *p* < 0.001) and lower subnetwork connectivity between FPN/DAN and LIM (B = -0.02, *p =* 0.019) and between the FPN/ DAN and VIS subnetwork (B = -0.027, *p =* 0.007) compared with low scoring children.

**Table 2:** Functional brain outcomes in participants with high OCS (n = 133) versus controls (n = 1568)

		95% CI				
Outcome	B	-	+	*β*	*t*	*p*
**Global graph measures**						
Modularity (Q)	0.005	0.001	0.009	0.259	2.666	0.008
**Subnetwork connectivity**						
LIM-FPN/DAN	-0.012	-0.023	-0.002	-0.227	-2.328	0.020
VIS-FPN/DAN	-0.015	-0.028	-0.002	-0.224	-2.283	0.023
**Thalamic node graph measures**						
lPFthal within-module degree Z	-0.118	-0.226	-0.009	-0.207	-2.119	0.034

Note: Models were adjusted for age, sex and ethnicity, and behavioral and emotional problems. Regression estimates were bootstrapped using 10,000 permutations. Unadjusted p-values are shown. CI = Confidence Interval; LIM = Limbic Network; FPN / DAN = Frontoparietal Network / Dorsal Attention Network; VIS = Visual Network; lPFthal = lateral prefrontal thalamus node

### Posthoc analysis of CBCL-OCS sumscore

We found a significant association between CBCL-OCS and within-degree Z-score of the posterior parietal thalamus in the second model (B = -3.56E-02, t = -3.173, pFDR = 0.034).

We added the results in the Supplementary tables (Table S12-S15).

## Discussion

We studied the network characteristics and subnetwork functional connectivity associated with obsessive-compulsive symptoms (OCS) in 9–12 year old children from the population-based Generation R study. Specifically, we examined global network topology, between-subnetwork connectivity of five data-derived subnetworks that included thalamic nodes, and network participation of the thalamic nodes in relation with OCS. In our preregistered analyses, we found no OCS-related differences in network topology nor in subnetwork functional connectivity, both in the continuous analysis and ‘probable OCD’ analysis. However, when comparing children endorsing at least one OCS with controls to align with recent literature, we found that the ‘high OCS’ group had higher modularity, lower FPN/DAN-LIM and FPN/DAN-VIS connectivity and lower lateral prefrontal within-module degree Z-score compared with controls.

The discrepancy between the preregistered and posthoc analysis is striking. We performed the ‘high OCS’ analysis to better align with Alexander-Bloch et al., where resting-state characteristics were also compared between youths endorsing at least one symptom compared with controls (Alexander-Bloch et al., 2021). We expected that the stronger contrast between probable OCD children and symptom-free children would translate to the strongest functional differences. Since this was not the case, the discrepancy suggests phenotypic differences underlying the ‘probable OCD’ group and ‘high OCS’ group. Follow-up analyses comfirmed this by demonstrating that hand washing symptoms were the main driver of the associations, and ruled out group differences on demographic characteristics and other emotional or behavioural problems.

The higher modularity found in the high OCS group provides support for differences on the global network scale. Even though graph measures have not been reported in other population-based OCS studies, this is consistent with the widespread connectivity associations found in population studies (Alexander-Bloch et al., 2021). To our knowledge, no previous studies have reported directly on modularity in relation to OCS in the general population. Though sparse, clinical OCD studies found that modularity was lower in adult and pediatric OCD compared with healthy controls (Armstrong et al., 2016; Shin et al., 2014). Interestingly, we found an opposite association with the high OCS group. Phenotypically, the clinical studies better resemble our original case-control design comparing probable OCD children and symptom-free controls, where no association with modularity was found. This could suggest that the direction of the association between OCS and modularity is dependent on symptom profile. Clinical studies distinguishing symptom dimensions in the context of global network topology are needed to test this hypothesis. Alternatively, modularity may be associated with the severity of symptoms, since clinical OCD patients showing lower modularity are most severely affected, followed by the probable OCD and high OCS group respectively.

Frontoparietal and limbic dysconnectivity fits with previous findings in clinical OCD and within existing neural models of OCS (Gürsel et al., 2018; Shephard et al., 2021; Tikoo et al., 2020). OCD has been proposed to arise from dysfunction of cortico-striato-thalamo-cortical circuits such as the frontolimbic, cognitive and frontoparietal circuits (van den Heuvel et al., 2016). Frontolimbic dysfunction drives the dysregulated/excessive fear in response to OCD-related stimuli (Shephard et al., 2021). The frontoparietal network is responsible for executive functions important for adequate coordination of goal-directed behavior (Marek & Dosenbach, 2018). Reduced connectivity between these regions could reflect inadequate emotion-behavior coupling, for example manifesting as excessive hand washing in response to anxiety-provoking contamination cues. Children endorsing at least one symptom had lower within-module degree z-score of the lateral prefrontal thalamic node. This indicates that the connections of this node are more dispersed across brain modules related to high OCS compared with controls. The prefrontal thalamic node corresponds to the mediodorsal thalamus (Fan et al., 2016). Even though no studies have directly investigated thalamic network participation in relation to OCD, altered thalamocortical connectivity of the mediodorsal nucleus has been reported in specifically pediatric OCD (Fitzgerald et al., 2011) and more recently in subclinical OCS (Suñol et al., 2021). Interestingly, Suñol et al. found evidence for symptom dimension-specific mediodorsal connectivity differences compared with controls not having symptoms (Suñol et al., 2021). Our findings also show a degree of overlap with connectivity studies on repetitive behaviors in other developmental disorder, which is further discussed in section S6.

We aligned with Pagliaccio et al. by conducting a posthoc analysis using the CBCL-OCS rather than the SOCS. However, given that the CBCL-OCS has several generic items that do not particularly pertain to OCS we draw more confidence from the analyses using the SOCS.

We previously reported structural thalamic differences related to probable OCD (Weeland et al., 2021). Given that the thalamus is an important hub region (van den Heuvel & Sporns, 2011), we hypothesized that structural alterations may also affect global network topology and the participation of specific thalamic subregions within the brain networks. Our results suggest that despite morphological differences in the thalamus, no functional network alterations are observed in probable OCD, but are confined to the high OCS group and the washing dimension in particular. This could reflect a difference in OCS severity. Morphological thalamus differences have primarily been reported in clinical OCD studies (Weeland Kasprzak, et al., 2022), while population-based studies found that OCS-related functional differences are not associated with thalamus morphology (Alexander-Bloch et al., 2021; Pagliaccio et al., 2021; Suñol et al., 2021). The structure-function relationship in the brain is complex and remains heavily debated (Suárez et al., 2020). Comparative studies between structural and functional connectivity have produced mixed results and demonstrate imperfect correspondence: at best half of the variance in functional connections is explained by structure (Suárez et al., 2020). In the literature, co-occurring alterations in thalamus morphometry and network topology have only been reported in epilepsy (Garcia-Ramos et al., 2017; Lee et al., 2020).

Confidence can be drawn from these findings for several reasons. We applied a rigorous imaging and pre-processing (including denoising) protocol to ensure the quality of the resting-state data (White et al., 2018). We preregistered our analysis plan and hypotheses before conducting any analyses (https://osf.io/azr9c). Also, in the resting-state sample we found significant relationships of sex or IQ with network topology that have been previously reported (Satterthwaite et al., 2015; Tyan et al., 2017), demonstrating sufficient sensitivity to detect these associations. Despite these strengths, there are also some methodological considerations. We calculated graph metrics based on fully weighted (unthresholded) networks. This includes weaker connections that may be spurious, potentially adding noise to the graph. Previous studies have dealt with this by using thresholded networks, but this raises the probability of removing true connections that purvey important information about the network and in some instances may introduce false-positive between-group differences in network metrics(van den Heuvel et al., 2017). Fully weighted networks have this problem to a lesser extent. Furthermore, by using an atlas-based approach we grouped voxel values into nodes, thereby averaging out the variation between voxels.

Future work should improve our understanding of the functional characteristics of OCD and OCS, in particular pertaining to children and thalamic connectivity. The ENIGMA-OCD working group is in preparation of a large-scale mega-analysis of resting-state functional MRI related to OCD across different age groups, which will partly solve the methodological restrictions of individual studies. A more thorough assessment of symptom dimensions could further disentangle the symptom-specific functional characteristics of OCS. To overcome the gap between structure and function, network characteristics of OCD could be assessed using structural connectivity or structural covariance analyses. A structural covariance analysis of ENIGMA-OCD already demonstrated lower small-worldness, modularity and clustering related to OCD in adults and adolescents (Yun et al., 2020).

## Conclusion

To summarize, thalamic morphology differences related to OCS in children do not translate to overall global network topological differences. Rather, children endorsing at least one symptom show differences in modularity, subnetwork connectivity and prefrontal thalamus network participation. Drawing from our results and existing literature, functional network characteristics seem to show symptom-specificity and may depend on the severity of OCS.

## Electronic supplementary material

Below is the link to the electronic supplementary material.

Supplementary Material 1

Supplementary Material 2

Supplementary Material 3

Supplementary Material 4

Supplementary Material 5

## Data Availability

Data from this study are available upon reasonable request to the director of the Generation R Study (generationr@erasmusmc.nl), subject to local, national and European rules and regulations.

## Declarations

*Author contributions*.

Author contributions included conception and study design (All authors), data collection or acquisition (CW), statistical analysis (CV, CW), interpretation of results (CW, CV, OH, TW), drafting the manuscript work or revising it critically for important intellectual content (CW, CV, OH) and approval of final version to be published and agreement to be accountable for the integrity and accuracy of all aspects of the work (All authors).
