# Supplementary material for: Obsessive-compulsive symptoms and resting-state functional characteristics in pre-adolescent children from the general population
Source: Brain Imaging Behav. 2022 Nov 2;16(6):2715–24. doi: 10.1007/s11682-022-00732-8 (PMC9712396; doi:10.1007/s11682-022-00732-8)
Supplement: Supplementary file 4 — Supplementary Material 4 [file 11682_2022_732_MOESM4_ESM.pdf]

Author Declaration Form
Brain Imaging and Behavior

Manuscript ID number (*if available*): BIOR-D-22-00085

Title: Obsessive-compulsive symptoms and resting-state functional characteristics in pre-adolescent children from the general population

Name of **First Author**: Cees Weeland

E-mail address of First Author: c.j.weeland@amsterdamumc.nl

Name of **Corresponding Author**: Cees Weeland

E-mail address of Corresponding Author: c.j.weeland@amsterdamumc.nl

AUTHORSHIP

I, the undersigned author(s), certify that:

1. I have read and approved the final version of the manuscript.
2. I have made substantial contributions to the submitted work, which may include study design, data acquisition and/or analysis, and data interpretation.
3. I have made significant contributions to the preparation of the manuscript and/or critical revisions for important intellectual content.
4. I will be accountable for all aspects of the submitted work, or I am accountable for the following specific aspects of the submitted work:

   ____

5. I agree to help investigate and resolve any issues/questions that may arise regarding the accuracy and integrity of the submitted work.

I understand that if necessary, the Editor-in-Chief of *Brain Imaging and Behavior* or designate may request deidentified data that has been submitted as part of the manuscript. In this event, I agree to produce the data.

I certify that this manuscript is not under consideration for publication in any other journal, nor has it been accepted for publication in any form, and no rights have been assigned to a third party.

I certify that all individuals who have made specific contributions to this manuscript but who do not fulfill the authorship criteria are listed with their specific contributions in the Acknowledgments section of the manuscript.

Author Declaration Form

Brain Imaging and Behavior

All funding sources directly or indirectly supporting this research have been acknowledged within the manuscript, including grant numbers where appropriate.

I understand that after the initial submission, authorship changes are strongly discouraged unless clearly warranted. If a major revision is submitted and authors have been added, removed or reordered, a rationale must be clearly indicated on the authorship change form, and all authors, prior and newly proposed, must sign the form indicating their agreement. Original signatures are required. Changes are subject to approval by the Editor-in-Chief.

CONFLICT OF INTEREST AND FINANCIAL DISCLOSURE

Within this manuscript, I agree to disclose any potential conflicts of interest, financial or otherwise, that might be perceived as influencing the objectivity of my work.

⇒ Potential sources of conflict include but are not limited to patents, copyrights, royalties, or stock ownership, membership on a company's board of directors, membership on a company's advisory board, and consultancy or speaker's fees from a company.

I understand that the conflict of interest section is to be placed at the end of the manuscript and may be stated as shown in the following example:

> John Doe currently receives royalties from pharmaceutical company X for the use of his patent.
>
> Jane Doe has no conflicts of interest to report.
>
> If none of the authors have disclosures to report, a single line may be added that states, "None of the authors have a conflict of interest to declare."

By signing this document, I agree to accept full responsibility for the work submitted in the manuscript, including the accuracy and integrity of the data and data analyses.

Additionally, I assert that there are no conflicts of interest, either personal or institutional, that have compromised the integrity of the work reported in this manuscript.

Author's Signature	Author's Signature
________Tonya White, MD, PhD_______	______________________________
Author's Printed Name and Date	Author's Printed Name and Date

____

Author Declaration Form
Brain Imaging and Behavior

Author's Signature

____

Author's Printed Name and Date

____

Author's Signature

____

Author's Printed Name and Date

____

Author's Signature

____

Author's Printed Name and Date

____

Author's Signature

____

Author's Printed Name and Date

____

Author's Signature

____

Author's Printed Name and Date

____

Author's Signature

____

Author's Printed Name and Date

____

Author's Signature

____

Author's Printed Name and Date

____

Author's Signature

____

Author's Printed Name and Date

____

Please note: A completed and signed copy of this form must be included with the submission. If authors are in multiple locations, more than one form may be used and submitted with the manuscript.
